# Factors associated with unintended pregnancy among women attending antenatal care in Maichew Town, Northern Ethiopia, 2017

**DOI:** 10.1186/s13104-019-4419-5

**Published:** 2019-07-05

**Authors:** Eskeziaw Abebe Kassahun, Liknaw Bewket Zeleke, Amanuel Addisu Dessie, Bisrat Gebrehiwot Gersa, Hayat Ibrahim Oumer, Hunegnaw Alemaw Derseh, Mulugeta Wodaje Arage, Getnet Gedefaw Azeze

**Affiliations:** 1 Department of Midwifery, Faculty of Health Sciences, Woldia University, Woldia, Ethiopia; 2 College of Medicine and Health Sciences, Debre Markos University, Debre Markos, Ethiopia; 3 Department of Public Health, Faculty of Health Sciences, Woldia University, Woldia, Ethiopia; 4 Minilik Hospital, Addis Ababa, Ethiopia; 5 Eman Herbal Center, Addis Ababa, Ethiopia; 6 Department of Nutrition, College of Medicine and Health Sciences, Bahir Dar University, Bahir Dar, Ethiopia

**Keywords:** Unintended pregnancy, Pregnant women, Maichew, Ethiopia

## Abstract

**Objective:**

Unintended pregnancy is one of the most public health issues in the world, and it is the major sexual and reproductive health problem which carries a higher risk of morbidity and mortality for women, often due to unsafe abortion. Even though family planning services are effective and available than ever before, unintended pregnancy and unsafe abortion are the major public health problems in the study area. Therefore, this study aimed at assessing the magnitude and associated factors of unintended pregnancy among pregnant women attending antenatal care follow up in Maichew town, northern Ethiopia. An institution based cross-sectional study was conducted on 329 pregnant women selected with a systematic sampling technique from April 5 to May 4, 2017.

**Result:**

The magnitude of unintended pregnancy among pregnant women attending antenatal care in Maichew was found to be 29.7% (95% CI 24.30, 35.50). On the other hand, single in marital status (AOR = 38.6, 95% CI 10.07, 148.01), living alone (AOR = 9.9, 95% CI 1.80, 53.40) and having three or four children (AOR = 3.5, 95% CI 1.10, 11.04) were factors associated with an unintended pregnancy. Creating awareness about unintended pregnancy associated factors and implication of unintended pregnancy is highly recommended.

## Introduction

Unintended pregnancies are pregnancies that are unwanted which occurs with no desire to have a child, mistimed which occurs before the desired time or unplanned at a time of conception [1]. Worldwide, an estimated 33 million unintended pregnancies are a result of contraceptive failure or incorrect use [2]. In developing countries, the majority of unintended pregnancy occurs due to using traditional family planning methods or not using any type of modern contraceptives [3], and it’s the main reason for induced abortion [4]. In Ethiopia, 24.7 and 42% of unintended pregnancy caused by contraceptive failure and not using contraceptive methods, respectively [5].

Globally, each day about 100 million sexual intercourse takes place, of these 1 million conceptions occur [6]. Among 208.2 million pregnancies of the world, 41% are unintended [7]. In addition, 213 million pregnancies occur each year, 89% of this occurs in developing countries, and 40% of these are unintended pregnancies [8].

Unintended pregnancy is the major sexual and reproductive health problems that impose to substantial health, economical and psychosocial costs to individual and society as well as significant emotional distress to women, families, and society [3, 9, 10]. Besides, contributing to late antenatal care (ANC) visit, increase exposure to the substance, less care for their child, and experiencing physical and psychological violence [11]. The impact of unintended pregnancy is higher during the adolescent period that levy to dropping out of school, unstable and lack of proper management of family relationships [12]. In addition, children born to teenage mothers are much more likely to experience a range of negative outcomes in later life, such as developmental disabilities, behavioral issues and poor academic performance [13].

About half of unintended pregnancies in developing countries result in unsafe abortion which accounts for 13% of maternal deaths [14]. Poor educational status, lack of access to health services and health education, poor economic status, single in marital status, peer pressure, sexual violence, and family planning failure were exposed to unintended pregnancy [9, 15, 16].

Unintended pregnancy is one of the most evident for the violation of women’s sexual and reproductive rights in developing countries. Despite the availability of highly effective methods of contraception, different studies in Ethiopia revealed that there is a high level of unintended pregnancy [17–20]. Therefore, this study aimed at assessing the magnitude and associated factors of unintended pregnancy among pregnant women attending antenatal care in Maichew town, northern Ethiopia.

## Main text

### Methods

#### Study design and period

An institution based cross-sectional study was conducted from April 5 to May 4, 2017.

#### Study area

The study was conducted in Maichew town, Tigray region, northern Ethiopia. Maichew town is found 665 km far from Addis Ababa, the capital of Ethiopia. According to the Central Statistical Agency of Ethiopia (CSA, 2007), the total population of 23,395 lives in Maichew town, of which 12,395 are females. The town has one general hospital, two health centers, and five private clinics.

#### Study population

All pregnant women who had ANC follow up during data collection were included in the study. However, pregnant women who were seriously ill during data collection were excluded.

#### Sample size determination and sampling procedure

The required sample size was determined using a single population proportion formula by considering the proportion of unintended pregnancy in Welkaite, 26.4% [17], a 95% confidence interval (CI) and 5% of margin of error. By adding 10% for the non-response rate, the final sample was 329 women. The data were collected from all governmental health institutions in Maichew town. Based on the previous 2 months of the Maichew Town Health Administrative report, 325, 189 and 125 pregnant women had ANC follow up in Lemlem Karl hospital, Maichew health center and Semere Melese health center, respectively. Then, 167 women from Lemlem Karl hospital, 68 women from Maichew health center, and 97 women from Semere Melese health center were selected using systematic sampling and proportional allocation technique.

#### Data collection tools and techniques

A structured interviewer-administered face to face interview was used to collect data. The questionnaire was consisted of sociodemographic and economic characteristics, sexual behaviors, and behavioral characteristics of participants which was initially prepared in English and translated into local languages (Amharic and Tigrigna) and back-translated to the English language by three language experts to check the consistency. A total of three BSc. Midwifery data collectors involved in the study. Data collectors received 3 days of training prior to data collection. A pretest was done on five percent of the sample on the Garjale health center (outside the study area). Finally, the collected data were checked for its consistency and completeness in each day before compiling.

#### Data processing and analysis

The collected data were checked and entered into EpiData version 3.10 and exported into SPSS version 20 statistical software for analysis. Descriptive statistics were carried out and the result was presented in text and table. Bivariate logistic regression analysis was used to identify factors associated with an unintended pregnancy. Crude odds ratio (COR) and adjusted odds ratio (AOR) with 95% confidence interval (CI) was calculated to identify factors associated with unintended pregnancy. Variables with a p-value less than 0.2 in the bivariable logistic regression analysis were retained into a multivariable logistic regression analysis to control the potential confounders. In the multivariable logistic regression analysis, variables with p-value < 0.05 at 95% confidence interval were considered as statistically significant.

### Result

#### Socio-demographic and economic characteristics

A total of 313 pregnant women participated in the study making the response rate 95.14%. The median age of the respondents was 26 years with an inter-quartile range (IQR) of 8 years. The majority (78.3%) of women were in the age range of 18–34 years. Nearly two-thirds (68.1%) of women lived in an urban area. Moreover, 33.1, 11.8 and 8% of women had no formal education, lived alone and had five or more children, respectively (Table 1).

**Table 1:** Socio-demographic and economic characteristics of pregnant women attending antenatal care in Maichew town, northern Ethiopia, 2017 (n = 313)

Variables	Frequency	Percentage
Residence		
Urban	213	68.0
Rural	100	32.0
Marital status		
Married	245	78.3
Single	68	21.7
Age (years)		
< 18	14	4.5
18–34	260	83.1
> 35	39	12.5
Mother’s education		
No formal schooling	97	31.0
Primary school	46	14.7
Secondary school	93	29.7
College or university	77	24.6
Mather’s occupation		
Student	56	17.9
Daily laborer or unemployed	37	11.8
Farmer	34	10.9
Civil servant	57	18.2
Merchant	27	8.6
Housewife	102	32.6
Husband’s education		
No formal education	102	32.6
Primary school	50	16.0
Secondary school	66	21.1
College or university	95	30.4
Husband’s occupation		
Student	34	10.9
Daily laborer or unemployed	28	8.5
Farmer	96	30.7
Civil servant	65	20.8
Merchant	90	28.8
Lived with		
Husband	214	68.4
Parents	62	19.8
Alone	37	11.8
Economic status		
Poor	159	51.0
Medium	107	34.0
Rich	47	15.0
No children		
0–2	212	68.0
3–4	76	24.0
≥ 5	25	8.0
HIV status		
Negative	298	95.2
Positive	10	3.2
Unknown	5	1.6

#### Sexual and behavioral characteristics

About, 18.8% of women had multiple sexual partners. A substantial proportion of women (78%) discussed with their partner on pregnancy-related issues. The most (93.3%) and 53% of women heard about and reported that school was a major source of information on contraceptive methods, respectively. Nearly one-third (65.8%) of the participants had good knowledge of contraceptive methods (Table 2).

**Table 2:** Sexual and behavioral characteristics of study participants in Maichew town, northern Ethiopia, 2017 (n = 313)

Category	Frequency	Percentage
Number of sexual partners		
One	254	81.2
Two and more	59	18.8
Communicate with partner on pregnancy		
Yes	244	78.0
No	69	22.0
Heard about family planning		
Yes	292	93.3
No	21	6.7
Source of information on family planning (n = 292)		
Family	50	17.1
Friend	121	41.4
School	155	53.1
Religious institution	13	4.5
Media	98	33.6
Health institution	117	40.1
Awareness of contraceptive methods		
Pills	255	87.3
Condom	171	58.6
Injectable	262	89.7
IUCD	183	62.7
Implant	92	31.5
Female sterilization	39	13.4
Male sterilization	33	11.3
Ever used family planning		
Yes	238	76.0
No	75	24.0
Used family planning^a^		
Pill	117	49.2
Condom	36	15.1
Injectable	165	69.3
IUCD	2	0.8
Implant	41	17.2
Knowledge of family planning		
Good	206	65.8
Poor	107	34.2

^a^Multiple response

#### Prevalence of unintended pregnancy 93012

The overall prevalence of unintended pregnancy was 29.7% (95% CI 24.30, 35.50).

#### Factors associated with unintended pregnancy

Both bivariable and multivariable logistic regression analyses were done to see the effects of the selected characteristics on unintended pregnancy. As it is presented in Table 3, knowledge of family planning, economic status, participant’s age, living arrangements, number of children, mother’s occupation, husband’s occupation, and marital status were factors associated with an unintended pregnancy in the bivariable analysis.

**Table 3:** Bivariate and multivariable logistic regression analyses of unintended pregnancy among pregnant women attending antenatal care in Maichew town, northern Ethiopia, 2017

Category	Unintended pregnancy		COR (95% CI)	AOR (95% CI)
	Yes	No		
Knowledge of contraceptive methods				
Poor	46	61	2.6 (1.68, 4.50)	1.3 (0.25, 6.70)
Good	47	159	1.0	1.0
Economic status				
Poor	66	93	4.8 (1.94, 12.0)	3.5 (0.60, 18.90)
Medium	21	86	1.7 (0.62, 4.44)	2.3 (0.40, 10.60)
Rich	6	41	1.0	1.0
Age of participant (years)				
< 18	11	3	1.0	1.0
18–34	64	196	0.09 (0.24, 0.32)	1.0 (0.09, 11.70)
> 35	21	18	0.23 (0.06, 0.90)	5.6 (0.30, 83.50)
Lived with				
Husband	29	185	1.0	1.0
Parents	33	29	7.3 (3.80, 13.60)	2.3 (0.70, 6.90)
Alone	31	6	32.8 (12.60, 85.80)	9.9 (1.80–53.40)*
Number of children				
0–2	62	150	1.0	1.0
3–4	20	56	0.9 (0.47, 1.55)	3.5 (1.10, 11.04)*
> 5	11	14	1.9 (0.81, 4.41)	2.6 (0.50, 13.10)
Mother’s occupation				
Student	21	81	1.0	1.0
House wife	34	22	6.0 (2.9, 12.24)	0.5 (0.08, 3.80)
Daily labor or unemployed	17	15	3.7 (1.63, 8.16)	1.7 (0.22, 13.40)
Peasant	8	26	1.2 (0.47, 2.99)	1.05 (0.11, 10.10)
Civil servant	6	51	0.45 (0.17, 1.2)	0.2 (0.02, 2.28)
Merchant	6	21	1.1 (0.39, 3.07)	1.05 (0.10, 8.10)
Husband’s occupation				
Peasant	33	63	1.0	1.0
Student	12	22	2.4 (1.22, 4.8)	1.3 (0.35, 4.80)
Daily laborer or unemployed	7	21	8.5 (3.49, 20.5)	1.1 (0.20, 6.20)
Civil servant	15	50	1.5 (0.50, 4.20)	1.3 (0.23, 6.60)
Merchant	16	74	1.3 (0.60, 3.10)	4.4 (0.10, 17.50)
Marital status				
Married	33	212	1.0	1.0
Single	60	8	48.1 (21–109.8)	38.6 (10.07, 148.01)*

*Significantly associated factors at p-value less than 0.05

Moreover, the result of multivariable logistic regression analysis revealed that marital status, number of children and living arrangement were significantly associated with unintended pregnancy. In this study, the high odds of unintended pregnancy were observed among women who lived alone (AOR = 9.9, 95% CI 1.80, 53.40) and had three or four children (AOR = 3.5, 95% CI 1.10, 11.04). Moreover, the odds of unintended pregnancy were 38.6 times (AOR = 38.6, 95% CI 10.07, 148.01) as high among women who were single compared to married (Table 3).

### Discussion

This study was conducted to assess the prevalence and associated factors of unintended pregnancy among women attending antenatal care in Maichew town, northern Ethiopia.

The prevalence of unintended pregnancy among women attending ANC in Maichew town was found to be 29.7% (95% CI 24.3%, 35.5%). The finding is in line with other studies done in Hosanna (34%) [21], Walkait (26%) [22] and Mekelle town (28.6%) [23]. But compared to other studies, this finding is lower than those of studies done in Addis Ababa (37%) [3], Kenya (41%) [24], and Wolaita zone (36.6%) [25]. The possible reason for the difference might be due to the socio-demographic difference of participants, particularly the aforementioned studies were conducted in capital cities whereas the current study was conducted in district town having poor awareness of family planning, and sexual and reproductive health rights. Moreover, the prevalence of this study is higher than those of other findings in Arbaminch town (19.4%) [26], South Africa (21.6%) [27] and India (22.2%) [21]. This might be due to the difference in the educational status of the participants having more awareness of family planning methods. The difference in the availability and accessibility of family planning methods may contribute to the difference.

Pregnant women who were single and living alone were independently associated with an unintended pregnancy. Single women were more likely to develop untended pregnancy than a married one. This study is supported by studies done in Gelemso [9], Wolaita zone [25], Mulago hospital [28], Ghana [29] and Kenya [24]. This might be due to women who are single or living alone are prone to unsafe sexual acts because of parents or families are important to monitor and support the behavior, and sexual and reproductive health of women. Moreover, it might be due to social influence or bad taboos that prevent women to use family planning methods.

In this study, women who had three or more children were more likely to develop unintended pregnancy as compared to their counterpart. The same finding was observed in Felegehiwote, Bahir Dar [30], Gelemso [31], Hosanna town [21], rural Ghana [29] and Zambia [32] studies. This might be due to women having a large family size (children) spend time to take care of their children which may result in missing appointments and even delay to seek maternal health services.

### Conclusion

The prevalence of unintended pregnancy was higher in the study area which confirmed the major public health problem. Marital status, the number of children and living arrangement were significantly associated with an unintended pregnancy. Hence, creating awareness on sexual and reproductive health rights, increasing the accessibility of maternal health services for women are recommended to reduce unintended pregnancy.

## Limitation of the study

The major limitation of this study was the use of small study participants. It’s recommended to use a large sample size for future studies.

## Data Availability

Data supporting this finding can be found in the manuscript. All the data sets supporting the paper are available from the corresponding author on reasonable request.

## Abbreviations

ANC: antenatal care
AOR: adjusted odds ratio
CSA: Central Statistics Agency
CI: confidence interval
COR: crude odds ratio
EDHS: Ethiopian Demographic and Health Survey
IQR: inter quartile range
SPSS: Statistical Package for Social Sciences
